# The Hydrogen Bonding in the Hard Domains of the Siloxane Polyurea Copolymer Elastomers

**DOI:** 10.3390/polym16172438

**Published:** 2024-08-28

**Authors:** Ming Bao, Tianyu Liu, Ying Tao, Xiuyuan Ni

**Affiliations:** State Key Laboratory of Molecular Engineering of Polymers, Department of Macromolecular Science, Fudan University, Shanghai 200438, China; 19110440004@fudan.edu.cn (M.B.); 21110440018@m.fudan.edu.cn (T.L.); 20110440021@fudan.edu.cn (Y.T.)

**Keywords:** siloxane polyurea copolymer, thermoplastic elastomer, hard domains, hydrogen bonding, temperature-dependent IR spectra

## Abstract

For probing the structure–property relationships of the polyurea elastomers, we synthesize the siloxane polyurea copolymer elastomer by using two aminopropyl-terminated polysiloxane monomers with low and high number-average molecular weight (*M_n_*), i.e., L-30D and H-130D. To study the influence of the copolymer structures on the film properties, these films are analyzed to obtain the tensile performance, UV-vis spectra, cross-sectional topographies, and glass transition temperature (T_g_). The two synthetic thermoplastic elastomer films are characterized by transparency, ductility, and the T_g_ of the hard domains, depending on the reacting compositions. Furthermore, the film elasticity behavior is studied by the strain recovery and cyclic tensile test, and then, the linear fitting of the tensile data is used to describe the film elasticity based on the Mooney–Rivlin model. Moreover, the temperature-dependent infrared (IR) spectra during heating and cooling are conducted to study the strength and recovery rate of the hydrogen bonding, respectively, and their influence on the film performance is further analyzed; the calculated *M_n_* of the hard segment chains is correlated to the macroscopic recovery rate of the hydrogen bonding. These results can add deep insight to the structure–property relationships of the siloxane polyurea copolymer.

## 1. Introduction

The polymer elastomers can recover to the highest degree of strain after stretching for large strain, with a structure composed of soft chains as the reversible phase and cross-linked structures as the fixed phase. The formation of the fixed phase is contributed to by the chemical crosslinking, crystalline regions, and hard domains with the hydrogen bonding interactions [1,2]. The broadly used polymer elastomers include the styrene-butadiene rubber (SBR), ethylene propylene diene monomer (EPDM) rubber, polyolefin elastomer (POE), poly (ester-amine), and polyurethane elastomer that are determined by the phase structure [3,4]. The polyurethane or polyurea elastomers contain the soft domains composed of polyols or polyamines, and their hard domains consist of the chains reacting by isocyanates and extenders. Due to the low glass transition temperature (T_g_) and low surface energy, the polysiloxane is introduced to the polyurethane elastomers, endowing the materials with excellent flexibility at low temperatures and smooth surfaces. Recently, the thermoplastic polyurea (TPU) elastomer containing polysiloxane has received rising attention for its melt processing capability and wider working temperature range.

The properties of the siloxane polyurea elastomer are influenced by many factors such as polysiloxane structures [5,6], diisocyanate structures and contents [7], and the types of chain extenders [8]. As reported in the work, the siloxane polyurea copolymers were synthesized by using aminopropyl terminated polydimethylsiloxane (PDMS) as soft segments with rather high *M_n_* (10,800 g/mol and 31,500 g/mol). By comparison, the copolymer based on PDMS of higher *M_n_* displays higher elongation at break and ultimate tensile strength [5]. Sheth et al. prepared segmented polyurea copolymers with three chain extenders including ethylene diamine (EDA), 1,6-diaminohexane, and 2-methyl-1,5-diaminopentane. The results show that the copolymers with shorter and symmetric chain extenders (EDA) display higher strength and wider temperature ranges [8]. To expand the end uses of the siloxane polyurea elastomers, it is vital to further study the copolymer structure–property relationships.

The fixed phases of the TPU are constructed by the hard domains through the hydrogen bonding interactions. In the conventional TPU using polyol monomers, the single ligands of hydrogen bonding are primarily attributed to the urethane N-H group as proton donors and the C=O or ester O groups as the proton acceptors [9]. The hydrogen bonding is influenced by the chemical structures and contents of hard segments [10], chain extenders [11], and M_w_ of the soft segments, thus impacting the microphase structures and physical properties of the material. For instance, the fluoridated TPU elastomers were synthesized utilizing 4,4′-[2,2,2-trifluoro-1-(trifluoromethyl)-ethylidene] bisphenol as the chain extender. It was found that the hydrogen bonding strength increased with the higher fluorine content, causing enhanced microphase separation and decreased transmittance for the elastomers [11]. In addition to polyol monomers, when introducing amine-terminated oligomers, the synthesized TPUs have different hard domains [12,13,14]. In detail, the double N-H groups in urea segments result in the presence of both single and ligands bidentate in the hydrogen bonding, implying stronger hard domain cohesion [15]. Incorporating amine-terminated polysiloxane into the copolymer may enhance the hydrogen bonding interaction through urea groups, leading to pronounced microphase separation, higher T_g_, and better elasticity [16,17]. Further knowledge about the effects of the polysiloxane structure on the hydrogen bonding is crucial to preparing the copolymer elastomers with the controlled structures and properties.

In this work, the siloxane polyurea copolymer elastomers are synthesized by introducing two aminopropyl-terminated polysiloxane oligomers with low *M_n_* (L-30D) and high *M_n_* (H-130D). The effects of the copolymer structures on the film properties are further analyzed based on the performance including the tensile performance, transmittance of UV-vis spectra, cross-sectional topographies, and T_g_. The two thermoplastic elastomer films are characterized by transparency, ductility, and the T_g_ of the hard domains. Furthermore, the elasticity behavior of the copolymer films is studied by the strain recovery and cyclic tensile test, and the linear fitting based on the Mooney–Rivlin model is conducted to analyze the film elasticity. Moreover, the temperature-dependent IR spectra during heating and cooling are utilized to monitor the shifts of carbonyl peaks, revealing the effects of the copolymer structures on the strength and recovery rate of the hydrogen bonding.

## 2. Experimental Section

### 2.1. Materials

Aminopropyl-terminated PDMS (L-30D: *M_n_* is 2200 g/mol; H-130D: *M_n_* is 9800 g/mol) was purchased from Waker Inc. (Munich, Germany), which was dried at 110 °C for 2 h before use. Isophorone diisocyanate (IPDI, 99%), and dibutyltin dilaurate (DBTDL, 95%) were bought from Aladdin Reagents Co. Ltd. (Shanghai, China). 1,3-Bis(3-aminopropyl) tetramethyldisiloxane (APTMS, 96%) was obtained from Rhwan Chemical Co. Ltd. (Shanghai, China) 1,4-Butanediol (BDO, purchased from Sinopharm Chemical Reagent Co. Ltd. (Shanghai, China)), Tetrahydrofuran (THF, 99.5%, anhydrous) was bought from Adamas Reagents Co. Ltd. (Shanghai, China).

### 2.2. Synthesis of the Siloxane Polyurea Copolymer Elastomers

**Scheme 1:**
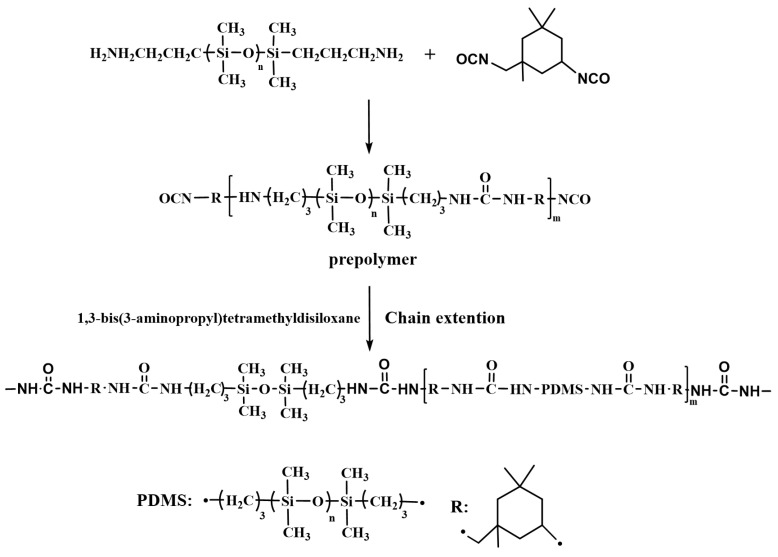
The schematic representation of the synthesis of siloxane polyurea copolymers using aminopropyl-terminated PDMS.

A series of siloxane urea copolymers using aminopropyl-terminated PDMS were synthesized through two steps, as illustrated in Scheme 1. Initially, the prepolymer was prepared by charging a mixture of PDMS oligomer (L-30D or H-130D) and THF into the four-necked round bottom flask, which was equipped with a nitrogen venting device. Then, the diluent containing IPDI and THF was added dropwise to the flask, followed by stirring the mixture for 2 h at room temperature. In the second step, the THF solution containing APTMS as the chain extender was dropped into the reacting mixture for four batches, with the mass contents of approximately 50%, 20% twice, and 10%, respectively. The reaction was allowed to proceed for 5 h, and the copolymer solutions with about 25% solid contents were obtained. For the copolymer synthesized using L-30D and H-130D, the mass contents of IPDI were set as 11%, 13%, and 18%, respectively, with their reactant compositions listed in Table 1.

### 2.3. Preparation of the Copolymer Elastomer Films

The copolymer solid products were prepared by casting the copolymer solutions into polytetrafluoroethylene molds at room temperature for 24 h, followed by drying at 50 °C overnight. The dried solid products were hot-pressed at 110 °C for 5 min, and the films about 1 mm thick were obtained.

### 2.4. Characterization

The Fourier transform infrared (FTIR) spectra of the reacting products were recorded using a Nicolet 6700 from Thermo Fisher Scientific Inc. (Waltham, MA, USA) in ATR mode with the diamond as the crystal on the attachment, covering the range from 4000 cm^−1^ to 525 cm^−1^ with a resolution of 4 cm^−1^. Meanwhile, the structure of the films was carried out in transmission mode. For the temperature scans, the thin films were placed in a heating cell that induced a hole to put the KBr tablet and maintain the transmittance. The heating rate was 5 °C per minute, followed by natural cooling at 20 °C. All measurements were conducted under a nitrogen atmosphere. The molecular weight of the copolymer was obtained using the gel permeation chromatography (GPC) on an Agilent 1206 instrument (Santa Clara, CA, USA). The samples were obtained by drying the copolymer solutions, and then solved in THF with the mass contents of 0.5%. The test was carried out using THF as the mobile phase with a flow rate of 1 mL/min.

The tensile tests were carried out using an Instron universal testing machine (Boston, MA, USA) with the specification of GBT 1040.3-2006. The dumbbell-shaped samples were utilized with the size of 50 mm × 4 mm × 1 mm, and the crosshead speed was 20 mm/min. Five samples were tested for each data point, and the average result was recorded. To evaluate the film resilience, the samples were stretched to strains of 300% or 200%. Then, one side of the samples was released from the clamp, followed by recording the strains versus time.

The cyclic tensile tests were performed at a maximum strain of 50% at a speed of 20 mm/min for 5 full cycles, with a 30 s rest between each cycle. The percentage of hysteresis was calculated by the curves of stress versus strain, as shown in the following formula [7].

(1) $$\%hystersis=\frac{A_{loading}-A_{recovery}}{A_{loading}}\times 100\%$$

where *A_loading_* is equal to the area increments under loading curves, and *A_recovery_* represents the area under recovery curves in the same cycle.

Dynamic mechanical analysis (DMA) was conducted using a QC800 analyzer from TA Company (Newcastle, WA, USA). The experiments were measured with a small tension clamp, heating the samples from −123 °C to 150 °C at a heating rate of 3 °C/min and a frequency of 1 Hz.

The film transmittance was obtained by Lambda750 ultraviolet-visible light (UV-vis) spectrometer from PerkinElmer (Waltham, MA, USA), with the wavelength ranging from 350 nm to 800 nm. The cross-sectional topography was imaged by a Tescan (Oberkochen, Germany) LaB_6_ scanning electron microscope (SEM). The cross-sections of the samples were obtained by fracturing the films in liquid nitrogen. Before testing, the cross-sections were gold-plated.

## 3. Results and Discussion

### 3.1. The Structural Characterization of the Siloxane Polyurea Copolymer Films

**Figure 1:**
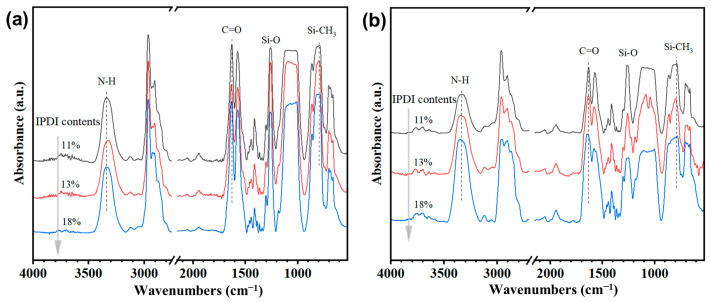
The FTIR spectra of the siloxane polyurea copolymer using L-30D (**a**) and H-130D (**b**).

Herein, the L-30D and H-130D oligomers were used to synthesize the siloxane polyurea copolymer, respectively. Scheme 1 gives the synthetic route of the copolymers. Figure 1a,b give the FTIR spectra of the copolymer films synthesized with L-30D and H-130D, respectively. The characteristic peaks of polysiloxane at 1263 cm^−1^ and 793 cm^−1^ are assigned to the in-plane bending vibration of the CH_3_ connecting with Si and the stretching vibration of Si-O, respectively [18], and the peaks at 2000 cm^−1^ can be attributed to the group of Si-CH=CH_2_, which might derive from the siloxane raw materials. For the copolymer films with the same monomer, the absorption peak intensities of the urea groups (3345 cm^−1^, 1630 cm^−1^, and 1570 cm^−1^) rise with the increasing IPDI contents. The results show that the urea group ratio in the copolymer increases with higher IPDI concentration. In addition, the results of NMR spectra for the oligomers and copolymers are shown in Supplementary Figure S1, which agrees with the FTIR results. By comparison, at the same IPDI content, the copolymer using H-130D displays higher relative peak intensities of the urea groups, reflecting more hard segment contents in the copolymer (Table 1). For the copolymer films using L-30D, the *M_n_* is 2.1 × 10^4^ g/mol (11% IPDI), 1.6 × 10^4^ g/mol (13% IPDI), and 1.6 × 10^4^ g/mol (18% IPDI), respectively, with a distribution index ranging from 1.4 to 1.7. At the IPDI contents of 11%, 13%, and 18%, the *M_n_* of the synthetic films using H-130D is 8.2 × 10^4^ g/mol, 7.8 × 10^4^ g/mol, and 5.2 × 10^4^ g/mol, respectively, with a distribution index between 1.7 and 1.8. In comparison with the copolymers using L-30D, the *M_n_* of the copolymers is significantly enhanced, which might be attributed to the introduction of more APTMS extenders to ensure the same IPDI contents. We can conclude that the siloxane polyurea copolymers have been synthesized.

#### 3.1.1. The Mechanical Properties of the Copolymer Films

**Figure 2:**
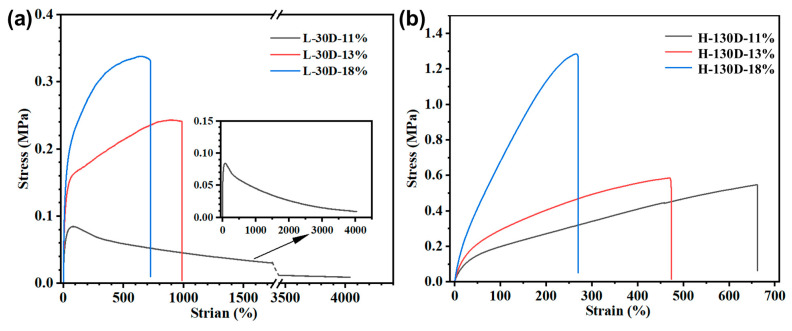
The tensile stress–strain curves of the copolymer films using L-30D (**a**) and H-130D (**b**).

The films were prepared from the synthetic copolymer products to study their mechanical performance, transmittance, and morphology. Figure 2a,b show the stress–strain curves of the films using L-30D and H-130D, respectively. When the L-30D is used as the soft segment, the films display excellent ductility with an elongation at break of more than 700%, which is higher than that of urea elastomer in the previous works [19,20,21]. Notably, the films with an IPDI content of 11% exhibit an elongation at break of more than 4000%. The tensile strength of the films ranges from 0.08 MPa to 0.33 MPa. As for the films using H-130D, with the IPDI content increasing, the elongation at break of the films decreases from roughly 670% to 250%, with the tensile strength enhanced from 0.6 MPa to 1.3 MPa. Compared with the copolymers containing L-30D, at the equal IPDI contents, the films using H-130D have much higher tensile strength, which indicates that the films using H-130D might have more physical cross-linked structures.

The areas under the stress–strain curves before fracture can represent the work of the tension (*W*), reflecting the energy absorption and toughness of the material [22,23]. For the films synthesized with L-30D, when the IPDI contents of the films are 11%, 13%, and 18%, the work of tension (*W*) is 0.15 N·m, 0.20 N·m, and 0.21 N·m, respectively. The work of tension (*W*) for the films using H-130D exhibits the values of 0.29 N·m, 0.16 N·m, and 0.26 N·m, respectively, when the IPDI contents of 11%, 13%, and 18%. Clearly, the H-130D-11% films exhibit the highest toughness.

**Figure 3:**
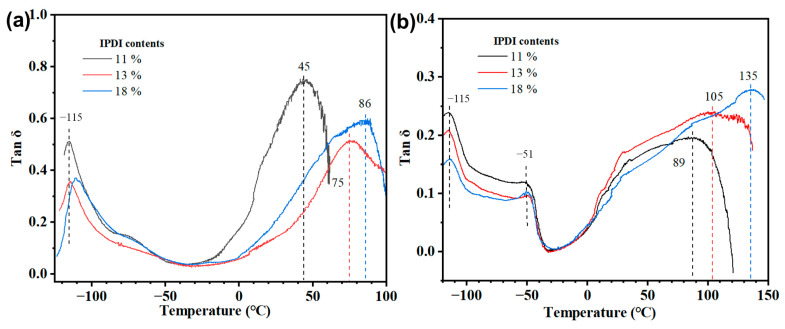
The loss factor (tan δ) versus temperature of the films using L-30D (**a**) and H-130D (**b**).

The T_g_ of the films can be analyzed through the DMA test. The results in Supplementary Figure S2 indicate that all samples have a rubbery plateau, followed by a rapid decline in the storage modulus with rising temperature, which agrees with the characterization of the thermoplastic elastomer. At the same IPDI content, the films using H-130D show a wider rubbery plateau, ranging from roughly −40 °C to around 70 °C (Supplementary Figure S2), indicating their wider working temperature ranges. The films have two T_g_ in total, as shown in Figure 3. The T_g_ at low temperature is approximately −115 °C, which is related to the soft segments of polysiloxane chains [7,8]. Another T_g_ at high temperature is ascribed to the hard domains. For the films using L-30D, the results in Figure 3a indicate that, when the IPDI content rises from 11% to 18%, the T_g_ of the hard domains increases from around 45 °C to 86 °C, which implies that the various IPDI contents can influence the hard domain structures. As shown in Figure 3b, the films using H-130D also display an increasing T_g_ of the hard domains ranging from 89 °C to 135 °C, as the IPDI content increases; moreover, the minor peaks at roughly −51 °C are attributed to the crystallinity of the polysiloxane that has high *M_n_*, which is close to the reported melting temperature for the polysiloxane crystalline region [8]. In compassion, there were no obvious peaks at around −50 °C for the copolymer using L-30D due to the poor crystallizing ability of the L-30D with low *M_n_*.

#### 3.1.2. The Transparency of the Copolymer Films

**Figure 4:**
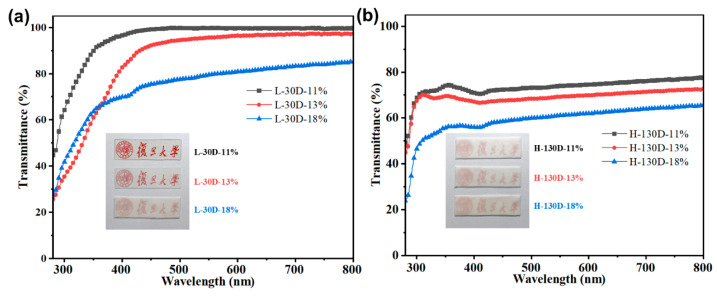
The UV-vis absorption spectra and photos of the films using L-30D (**a**) and H-130D (**b**). The characters covered by the films mean Fuand University in Chinese.

Figure 4a gives the UV-vis absorption spectra of the films synthesized with L-30D. When the IPDI contents are 11% and 13%, the films show quite high transparency at visible light, with light transmittance of roughly 99% and 96% at the wavelengths from 500 nm to 800 nm, respectively, which is similar to the transmittance of non-siloxane block polyurethane coatings described in the literature [24,25]. As the IPDI content increases to 18%, the films display a light transmittance ranging from 80% to 68%. As presented in Figure 4b, the films using H-130D display the transmittance below 80%. For the L-30D films using 11% and 13% contents of IPDI, the characters on the bottom of the films can be seen clearly, which agrees with the results of the higher transmittance. It is known that the transmittance is affected by the phase separation structure of the material containing multiple compounds [26,27]. In the siloxane polyurea copolymer, there is a notable difference in the polarity among the soft domains of polysiloxane and the hard domains formed by hydrogen bonding. The solubility parameters for the soft and hard domains are 15.6 (J·cm^−3^)^1/2^ and 45.6 (J·cm^−3^)^1/2^, respectively, showing a phase-separated trend [28]. Conclusively, the copolymer films exhibit decreased transmittance when the H-130D is introduced, implying that the films might have higher degree of the phase separation that leads to more light scattering or reflection.

**Figure 5:**
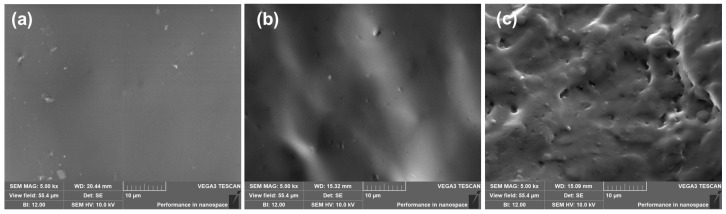
The cross-sectional topography of SEM for the films using L-30D, the IPDI contents: (**a**) 11%, (**b**) 13%, (**c**) 18%.

**Figure 6:**
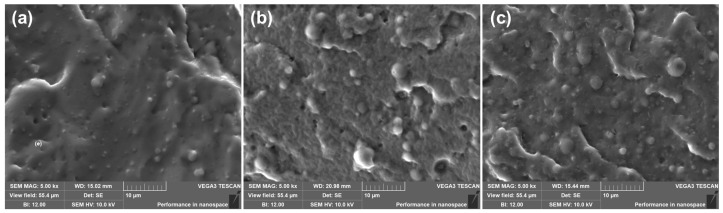
The cross-sectional topography of SEM for the films using H-130D, the IPDI contents: (**a**) 11%, (**b**) 13%, (**c**) 18%.

Furthermore, the SEM has been used to study the cross-sectional topographies of the synthetic films after brittle fracture in liquid nitrogen. Figure 5 images the cross-sectional topographies of the films using L-30D. When the IPDI content is 11%, the cross-section is flat and smooth; as the IPDI content increases to 13%, little particles appear on the cross-section. In contrast, when the IPDI content is raised to 18%, the topography changes distinctly, resulting in more obvious heterogeneous phase structures; specifically, little particles are distributed with the size from 1 μm to 2 μm. These results are consistent with the film transmittance test in Figure 4a. The SEM images in Figure 6 display the cross-sectional topographies of the films using H-130D. The results indicate that the films display various heterogeneous structures, characterized by multiple dispersed phases ranging scatter across the cross-sections, and the number of the dispersed phases increases with the higher IPDI content. By comparison, the thermoplastic copolymer films based on H-130D exhibit much lower transmittance and a higher degree of phase separation at the same IPDI content.

### 3.2. The Modeling Analysis of the High Elasticity for the Copolymer Films

**Figure 7:**
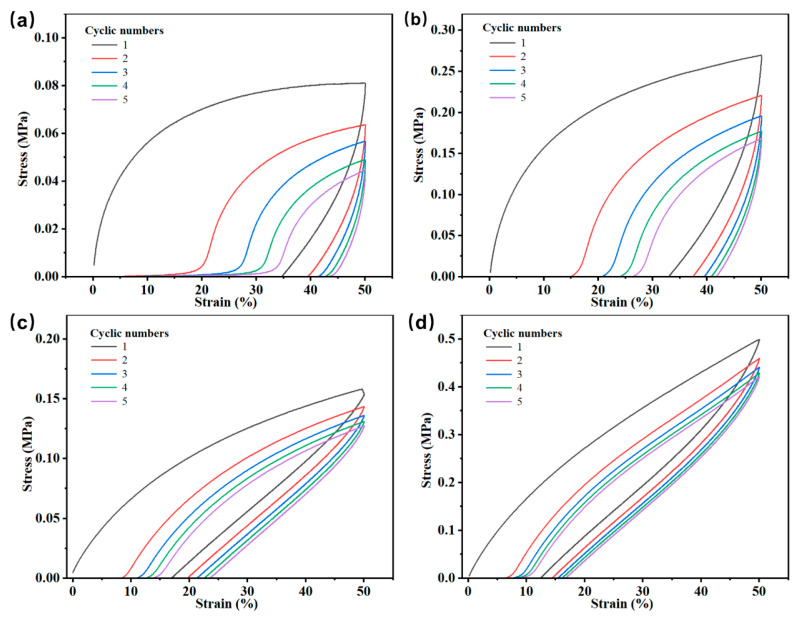
The cyclic tensile curves of the samples: (**a**) L-30D-11%, (**b**) L-30D-18%, (**c**) H-130D-11%, and (**d**) H-130D-18%.

The tensile recovery and cyclic tensile tests are utilized to characterize the elasticity of the copolymer films. The results in Supplementary Figure S3 indicate that the strain can recover to roughly 60% at the greatest level for the films using L-30D, while the films containing H-130D exhibit remarkable resilience with the ability to recover to less than 10% strain. The cyclic tensile curves are presented in Figure 7, with the hysteresis listed in Table 2. The hysteresis is calculated by the Formula (1) and represents the energy dissipation of the films. The results show that the films have the highest hysteresis in the first cycle, which is caused by the energy dissipation during the tensile process. The energy dissipation is primarily formed by the dissociation of the hydrogen bonding, the deformation of soft segments, and the interaction of the hard and soft segments [29,30]. It is seen that the film hysteresis tends to decrease with the increase of IPDI contents, indicating that the increasing IPDI contents may affect the film phase structure. Compared with the films using L-30D, the films containing H-130D display significantly lower hysteresis at the same IPDI contents, and their differences enlarge with the increasing IPDI contents. The result might be ascribed to fewer breakages of the hydrogen bonding owing to the hard domains connected by soft segments with higher *M_n_* [25].

As for the copolymer films using the two polysiloxane monomers, the results mentioned before show notable distinctions in many aspects, such as the limited highest temperature (T_g_), toughness (*W*), ductility, resilience, and the hysteresis of the cyclic tensile process. These results reflect the influence of the oligomer structures and isocyanate contents on the mechanical performance, thermal properties, and transparency of the copolymer films. Having realized that the copolymer properties are mostly affected by the hydrogen bonding interaction, we investigate the hydrogen bonding by the modeling fitting and the temperature-dependent IR spectroscopy to study the effect of the copolymer structures on the film properties.

For the ideal highly elastic materials that are isotropic in the undeformed state, the physical properties can be specified by the stored energy function W. Based on the Mooney–Rivlin model [29,30], the strain during the deforming process in a single direction can be described by the Formula (2).

(2) $$\delta =(\lambda -\frac{1}{\lambda^{2}})(2C+2k\frac{1}{\lambda })$$

where *λ* represents the strain in the tensile direction (*λ* ≠ 0), and *δ* is calculated through the *W* as a function of the strain invariants (*λ*). C and k are both the modeling parameters; the parameter *C* reflects the cross-linked degree of the material, and its value increases with the cross-linked degree.

The Formula (2) can be transformed according to the following formula:

(3) $$\left[f^{*}\right]=\delta /\left(\lambda -\frac{1}{\lambda^{2}}\right)=2C+2k\frac{1}{\lambda }$$

where [$f^{*}$] is named the contrastive strain.

**Figure 8:**
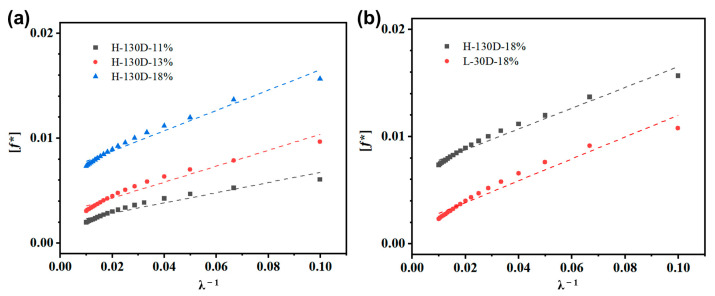
The scatters and fitted lines (dashes) of [*f*^∗^] versus *λ*^−1^ for the copolymer films using H-130D (**a**) and the films by the oligomers of different *M_n_* (**b**).

As for the copolymer films using H-130D, based on the tensile strain–stress data within the strain range from 10% to 100%, the contrastive strain [$f^{*}$] is calculated firstly, followed by obtaining the function curves of [$f^{*}$] and λ^−1^ with the [$f^{*}$] and λ^−1^ as Y-axis and X-axis, respectively, as shown in Figure 8. It is seen that the [$f^{*}$] and λ^−1^ approximately conform to the linear relation, which indicates that the Mooney–Rivlin model can describe the elasticity of the siloxane polyurea copolymer. Furthermore, the linear fitting is conducted according to the Formula (3), with the calculated model parameters of *C*, *k*, and R^2^ listed in Table 3. The R^2^ of all the samples is higher than 0.92, certifying the reliability of the fitting results.

The parameter 2C is the Y-intercept for the fitted lines of the [$f^{*}$] and *λ*^−1^. The results in Figure 8a indicate that the model parameter C is enhanced with the increase of IPDI contents. Based on the model, the value of C is positively related to the elastomer cross-linked degree, which indicates that the synthetic polyurea copolymer films display a higher cross-linked degree as the IPDI content increases. In the TPU materials, the physical crosslinking is mostly composed of the interaction of hydrogen bonding. Therefore, the changing trend of the parameter C indicates that the hydrogen bonding interaction is improved with the IPDI content increasing.

Moreover, at the same experimental conditions with the IPDI contents of 18%, the tensile data for the films using two oligomers separately are also fitted and calculated, with the results shown in Figure 8 and Table 3. At the same IPDI content, when using the oligomer of low *M_n_* (L-30D), the synthetic film displays a smaller parameter C, suggesting that the film displays fewer crosslinking due to the weaker hydrogen bonding interaction.

### 3.3. Analyzing the Hydrogen Bonding of the Hard Domains by Using Temperature-Dependent IR Spectroscopy

In the polyurea materials, the soft segments of the copolymer are typically composed of diamine oligomers, and the hard segments are formed by the chains synthesized by the isocyanate and extender. Due to the hydrogen bonding, the hard segments tend to aggregate as hard domains, resulting in the microphase separation structure. The FTIR spectra are an efficient tool for analyzing the hydrogen bonding interactions. For example, the formation of hydrogen bonding may cause a shift in the carbonyl peaks towards lower wavenumbers. According to the literature results [27,31], the polyurea copolymer contains three carbonyl states: free (~1670 cm^−1^), disordered (~1645 cm^−1^), and ordered (~1620 cm^−1^). The free carbonyl is without the hydrogen bonding, while the disordered and ordered carbonyls are formed by the hydrogen bonding of single and bidentate ligands, respectively.

**Figure 9:**
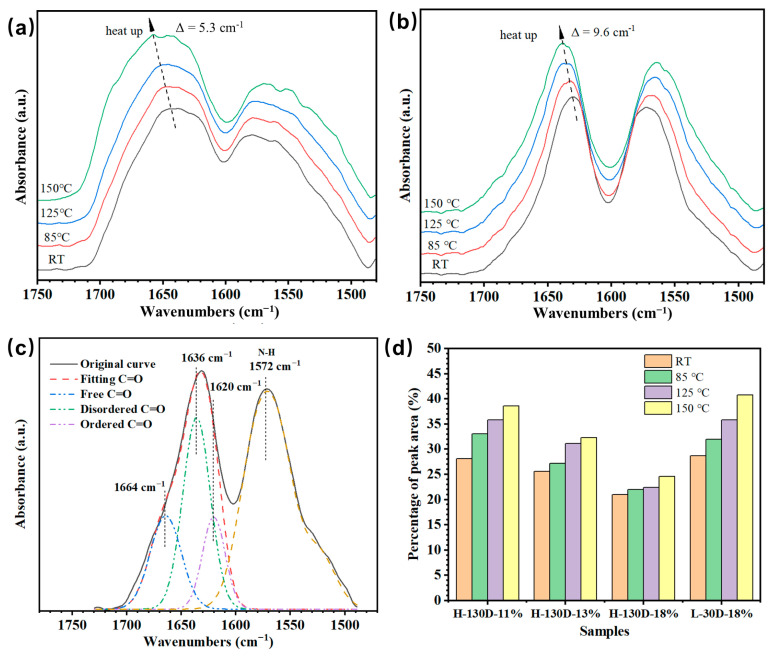
The FTIR spectra of the carbonyl during heating for the films using H-130D (**a**) and L-30D (**b**) at the IPDI content of 18%. (**c**) The fitting example of carbonyl peaks in IR spectra for the films. (**d**) The area percentage of free carbonyl peaks at rising temperatures for these samples.

The peaks in the IR spectra exhibit different wavenumbers for the carbonyl with or without the hydrogen bonding. Therefore, as the samples are heated, the weakening of hydrogen bonding can result in shifts in the carbonyl peak [32]. Based on this theory, when the films were heated from room temperature to 150 °C, the IR spectra were tested at various temperatures, with the change in carbonyl peaks presented in Figure 9 and Supplementary Figure S4. The carbonyl peaks in all copolymer samples exhibit a shift towards higher wavenumbers. The results suggest that the hydrogen bonding is dissipated upon heating, leading to an increasing amount of the free carbonyls.

Considering that the carbonyl peaks associated with three states are detected at various wavenumbers, the carbonyl peaks can be peak fitted, as shown in Figure 9c. These results reveal that the free carbonyl peaks in all samples rise in percentage to various extents during heating. Notably, at the same temperature, the free carbonyl contents decline as the IPDI contents increase, certifying that the carbonyl with hydrogen bonding is enhanced in percentage. Furthermore, with the same IPDI amount (18%), the temperature-dependent IR spectra were detected for the samples using different oligomers. For the copolymer containing H-130D, the free carbonyl percentage at the same temperature is much lower than that of the copolymer using L-30D. It implies that the copolymers containing H-130D have stronger hydrogen bonding interaction.

The experimental evidence has exhibited the various performances of the films using L-30D and H-130D, which are correlated to the hydrogen bonding strength of the hard domains. The stronger hydrogen bonding may produce more separated hard domains, implying that the films using H-130D display more structures of heterogeneous phase (Figure 5), which can explain the relatively low visible-light transmittance of the films (seen in Figure 4b). As reported, the microphase separation of hard segments generally forms the physical crosslinking as the fixed phase in the TPU materials [1,2]. Obviously, the films containing L-30D display less cross-linking owing to the weaker interaction of the hydrogen bonding, which results in lower tensile strength and T_g_, as well as better ductility with the elongation at break of more than 700%. Meanwhile, compared with the films containing H-130D, the films using L-30D show the visible stress yield within 100% strain (seen in Figure 2a), which may be attributed to the unstable hard domains due to the relatively few hydrogen bonding. Moreover, the more separated hard domains can contribute to the better toughness of the films with H-130D. Additionally, the toughness of the TPU is also correlated to the entanglement of polymer chains.

The deformation of the hard domains may affect the elastic recovery rate and degree of the films. Meanwhile, heating the films can disrupt the hard domains by dissipating the hydrogen bonding. To study the recovery ability of the hydrogen bonding, the temperature-dependent IR spectra are utilized to analyze the changing rule of carbonyl peaks during cooling. The variation of carbonyl peaks is closely associated with the relaxation ability of the hard segments.

**Figure 10:**
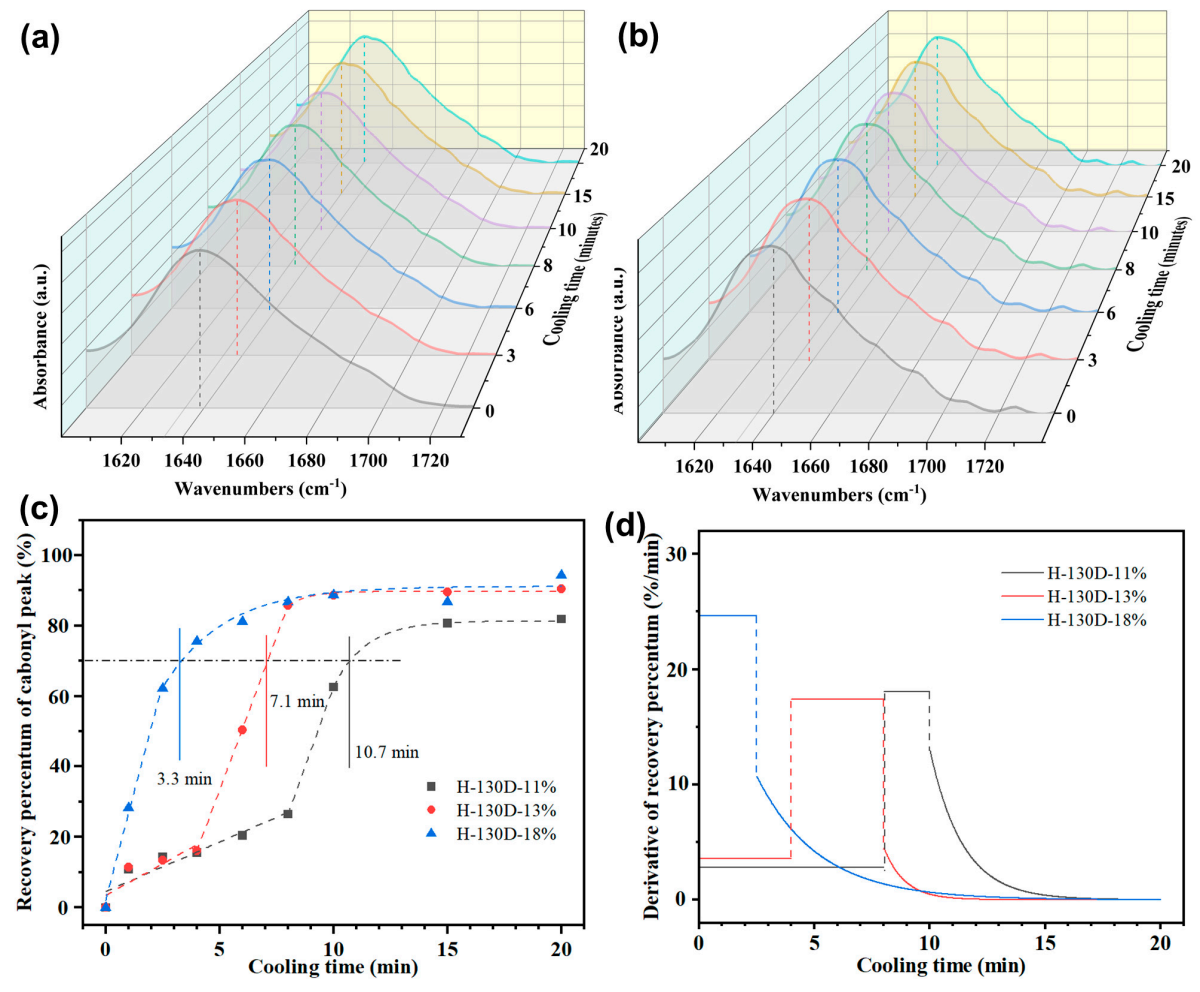
The FTIR spectra of the carbonyls during cooling for the films using H-130D, the IPDI contents: (**a**) 11%; (**b**) 13%. The recovery percentage curves (**c**) and the recovery rate (**d**) of the carbonyl peaks for the films using various IPDI contents.

**Figure 11:**
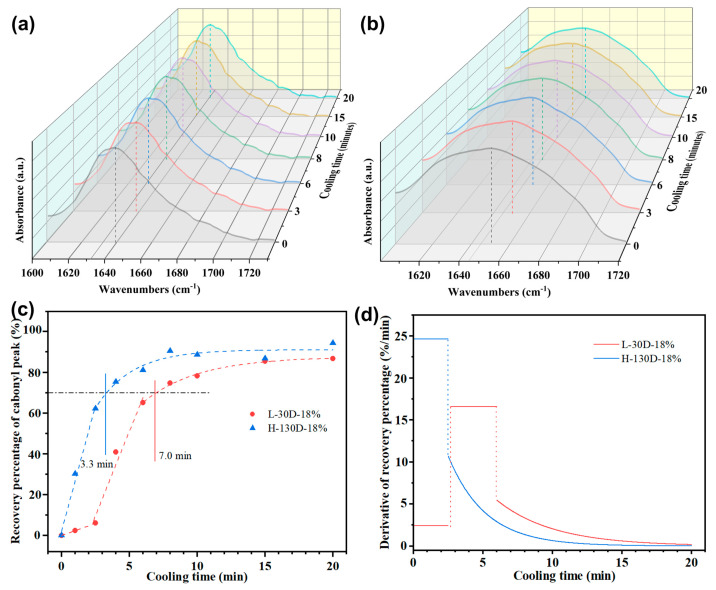
The FTIR spectra of the carbonyls during cooling for the films using L-30D (**a**) and H-130D (**b**). The recovery percentage curves (**c**) and the recovery rate (**d**) for the carbonyl peaks of these films.

The films were heated to 150 °C under the same condition and then cooled naturally. The IR spectra were recorded at various cooling times to observe the change in carbonyl peaks, as shown in Figure 10 and Figure 11. The results indicate that the carbonyl peak of each film shifts toward lower wavenumbers during cooling, implying the hydrogen bonding recovery. The recovery percentage (*φ*) of carbonyl peaks is defined using the following formula.

(4) $$\varphi =\frac{\lambda_{0}-\lambda_{t}}{\lambda_{0}-\lambda_{RT}}\times 100\%$$

where *λ*_0_, *λ*_t_ represent the wavenumbers of carbonyl peaks at the start of cooling and after t minutes of cooling, respectively, and *λ*_RT_ is the wavenumber of carbonyl peaks at room temperature. Herein, *φ* can qualitatively describe the recovery degree of hydrogen bonding.

Figure 10c presents the recovery percentage curves for the films using different IPDI contents, with the oligomer being H-130D. It is evident that *φ* increases with the higher IPDI contents, exhibiting more obvious differences at the beginning of cooling. Based on the time to need for reaching 70% (*φ* = 70%) of the original carbonyl peak (t_0.7_), the macroscopic recovery rate of the hydrogen bonding can be compared quantitatively. As shown in Table 4, the required time (t_0.7_) for the samples declines from 10.7 min to 3.3 min, as the IPDI content increases from 11% to 18%.

According to Formula (5) [6,33], we have calculated the ${\bar{M}}_{n}$ of the hard segment chains, i.e., ${\bar{M}}_{H}$.

(5) $${\bar{M}}_{H}=M_{S}\times \omega +2M_{0}$$

where *M_s_* represents the *M_w_* of the polysiloxane oligomers, and ω is the ratio of the mass of the hard segments consisting of isocyanates and extenders to the total mass of the reactants. *M*_0_ takes the value of 16 g/mol, which indicates *M_n_* of the amino (-NH_2_) group in the urea group at the two ends of the hard segment chain.

Table 4 presents the ${\bar{M}}_{H}$ of the polyurea copolymers synthesized with H-130D and various IPDI contents. It is observed that the ${\bar{M}}_{H}$ is negatively related to the t_0.7_, meaning that the required time of recovery declines with the higher ${\bar{M}}_{H}$. In theory, it is more possible to aggregate the polymer chains with higher *M_n_*. Therefore, ${\bar{M}}_{H}$ is a significant factor that influences the hydrogen bonding recovery.

To further study the hydrogen bonding recovery, the derivative of the *φ* against t curves is calculated to obtain the function relation of *φ*′ (*dφ*/*d*t) and cooling time, which represents the instantaneous recovery rate. As for the H-130D-18% film, the hydrogen bonding rapidly reverts during cooling, followed by a decrease in the recovery rate. Separately, the other copolymers containing H-130D display a very low *φ*′ within 8 min, indicating minimal hydrogen bonding recovery at the start of cooling; afterward, a relatively high constant rate is observed, followed by a gradual slowdown. Based on these results, the hydrogen bonding recovery can be categorized into three stages: the initial stage, with a very low *φ*′; the second stage, having a constant rate; and the third state, with a declining rate. The duration of the initial stage varies for each film, and the retention time for the H-130D-18% films can be regarded as zero. Figure 10d illustrates that when the IPDI contents increase from 11% to 18%, the sample retention time in the initial stage decreases from 7.2 min to 0 min. Furthermore, the recovery rates at the second stage are nearly identical. Based on the above results, it can be concluded that the notable difference in the initial stage of *φ*′ leads to variations in the recovery time (t_0.7_). The polymers with higher molecular weight have a greater tendency to aggregate, thereby the retention time at the initial stage should be influenced by the ${\bar{M}}_{H}$ for the copolymer. Moreover, the T_g_ also impacts the relaxation time of the polymer chains, indicating that the recovery of the hydrogen bonding may be influenced by both the hard segments and T_g_.

Additionally, at the same IPDI contents of 18%, the synthetic films using L-30D and H-130D were characterized by the temperature-dependent IR spectra during cooling. The relation curves of *φ*′ and t are analyzed as before, with the results shown in Figure 11. The copolymer films with L-30D require a longer recovery time, with a difference in t_0.7_ between the two samples of 4 min. Moreover, the calculated ${\bar{M}}_{H}$ of the L-30D copolymer is 701 g/mol, which is far less than that of the films using H-130D. The result still agrees with the negative correlation of the ${\bar{M}}_{H}$ and t_0.7_.

During the tensile process at large strain, the breakage of hydrogen bonding occurs in the deformation of hard domains, leading to energy dissipation [34]. Similar to the heating and cooling process, the dissipation and recovery of the hydrogen bonding occur during the cyclic tensile process, indicating the deformation and reorganization of the hard domains. For the films containing H-130D, the stronger hydrogen bonding and the faster recovery rate reflect less irreversible deformation of the hard domains, reflecting less energy dissipation (Table 2). The rule is also suitable for the films using the same oligomer with higher IPDI contents. Moreover, the film resilience is determined by the relaxation of polymer chains, which are mainly influenced by the recovery rate of the deformed hard domains. Evidently, the films using H-130D show faster recovery rates of the hydrogen bonding, consistent with their better resilience (Supplementary Figure S3).

## 4. Conclusions

We have synthesized the siloxane polyurea copolymer elastomers using two oligomers with low *M_n_* (L-30D) and high *M_n_* (H-130D) as soft segments, respectively. Further, the influence of the copolymer structures on the film properties is studied by the Mooney–Rivlin model and the temperature-dependent IR spectroscopy. The two kinds of thermoplastic elastomer films are characterized by ductility, transparency, and T_g_ of the hard domains, respectively. Moreover, the film transmittance is correlated to the phase structures.

The elasticity behavior of the copolymer films is studied by the strain recovery and cyclic tensile test, and the Mooney–Rivlin modeling fitting based on tensile data can describe the film elasticity, correlated to the degree of physical cross-linking. The results of the temperature-dependent IR spectra during heating show that the copolymers display stronger hydrogen bonding strength when using the higher diisocyanate contents or the oligomer H-130D, which can explain the influence on the film properties. Further, the IR spectra during cooling are utilized to analyze the hydrogen bonding recovery. For the copolymers using H-130D, the ${\bar{M}}_{H}$ is negatively related to the recovery rate of the hydrogen bonding, which is also suitable for the films using two various monomers at the same IPDI contents. The hydrogen bonding interactions can explain the distinctions of the copolymer film properties; specially, the lower hysteresis of the H-130D films is attributed to the strong hydrogen bonding and the great stability of the hard domains. These results can be helpful in designing other TPU materials.

## Figures and Tables

**Table 1:** The reactant compositions of the copolymer using aminopropyl-terminated PDMS.

Samples	PDMS [mmol]	IPDI [mmol]	APTMS [mmol]	IPDI Content [wt%]	^a^ Hard Segment content [wt%]
L-30D-11%	5.0	6.4	1.4	11	13
L-30D-13%	5.0	8.7	8.7	13	20
L-30D-18%	5.0	13.4	8.4	18	30
H-130D-11%	1.5	9.0	7.5	11	21
H-130D-13%	1.5	12.0	10.5	13	26
H-130D-18%	1.5	18.8	17.3	18	36

^a^ Hard segment content is the mass percentage of IPDI and APTMS among the total components.

**Table 2:** The hysteresis values of the cyclic tensile curves for the copolymer films.

Samples/Cycles	L-30D-11%	L-30D-18%	H-130D-11%	H-130D-18%
1	87.6	84.7	53.3	45.3
3	84.2	81.7	45.7	35.5
5	84.0	81.5	45.3	35.0

**Table 3:** The fitted parameters used for the Mooney–Rivlin model.

Samples	*C* (MP_a_)	*k* (MP_a_)	^a^ R^2^
L-30D-18%	1.0 × 10^−3^	0.018	0.95
H-130D-18%	3.5 × 10^−3^	0.049	0.97
H-130D-13%	1.4 × 10^−3^	0.038	0.96
H-130D-11%	1.0 × 10^−3^	0.025	0.92

^a^ R^2^ represents the linear regression index of the fitting curves.

**Table 4:** The average *M_n_* of hard segment chains and the required time for reaching *φ* = 70%.

Samples	${\bar{M}}_{H}$ [g/mol]	t_0.7_ [min]
H-130D-11%	2102	10.7
H-130D-13%	2651	7.1
H-130D-18%	3609	3.3

## Data Availability

The raw data supporting the conclusions of this article will be made available by the authors on request.

## Supplementary Materials

The following supporting information can be downloaded at: https://www.mdpi.com/article/10.3390/polym16172438/s1, Figure S1: The 1H-NMR of the samples: (a) the oligomer L-30D, (b) the oligomer H-130D, (c, d) the copolymer L-30D-11% and L-30D-18%; (e, f) the copolymer H-130D-11% and H-130D-18%. Figure S2: The storage modulus versus temperature of the films using L-30D (a) and H-130D (b). Figure S3: The strain recovery curves of the films using L-30D (a) and H-130D (b). Figure S4: The FTIR spectra of the carbonyl during heating for the films using H-130D with the IPDI contents of 11% (a) and 13% (b).
