# Supplementary material for: The Hydrogen Bonding in the Hard Domains of the Siloxane Polyurea Copolymer Elastomers
Source: Polymers (Basel). 2024 Aug 28;16(17):2438. doi: 10.3390/polym16172438 (PMC11397959; doi:10.3390/polym16172438)
Supplement: Supplementary file 1 [file polymers-16-02438-s001.zip › polymers-3153938-supplementary.pdf]

The Hydrogen Bonding in the Hard Domains of the Siloxane Polyurea Copolymer Elastomers

Ming Bao[a], Tianyu Liu[a], Ying Tao[a], Xiuyuan Ni[b*]

[a]State Key Laboratory of Molecular Engineering of Polymers, Department of Macromolecular Science, Fudan University, Shanghai, People's Republic of China;

[b]State Key Laboratory of Molecular Engineering of Polymers, Department of Macromolecular Science, Fudan University, Shanghai, People's Republic of China.

E-mail: xyni@fudan.edu.cn.

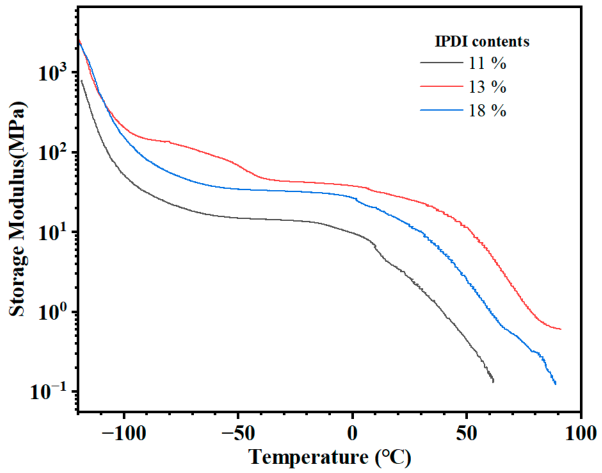

Figure S1: The storage modulus versus temperature of the films using L-30D.

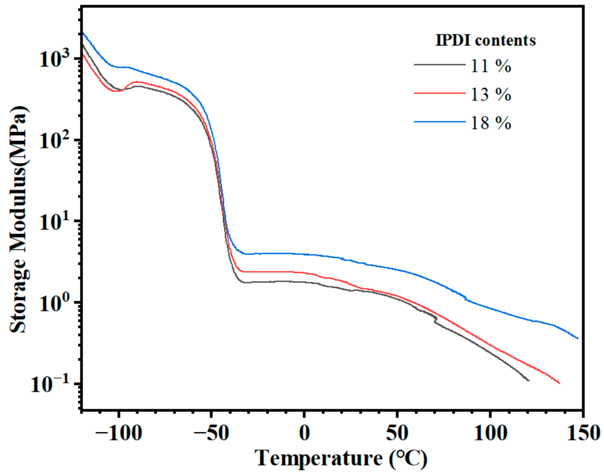

Figure S2: The storage modulus versus temperature of the films using H-130D.

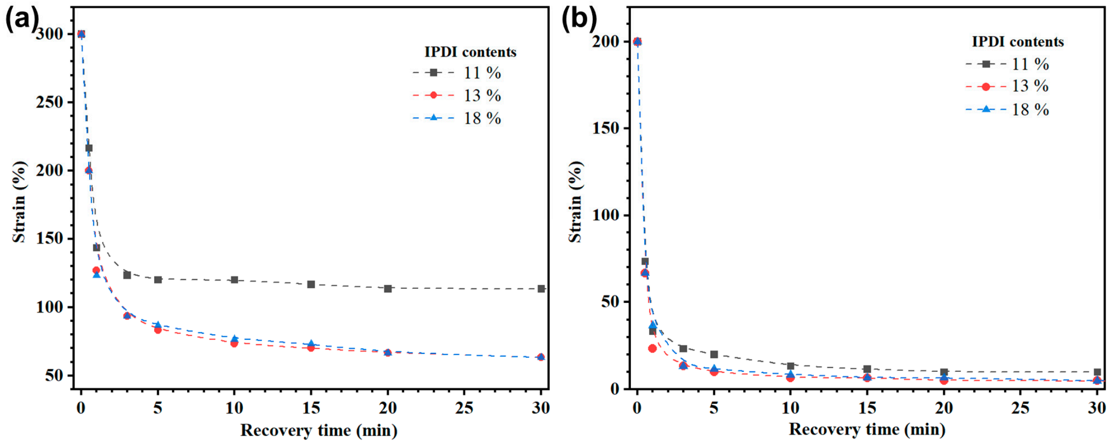

Figure S3: The strain recovery curves of the films using L-30D (a) and H-130D (b).

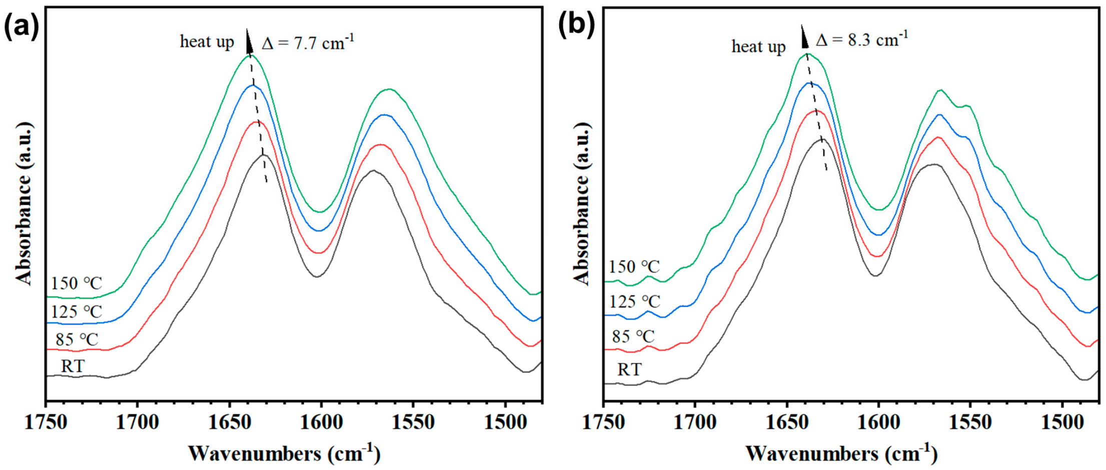

Figure S4: The FTIR spectra of the carbonyl during heating for the films using H-130D with the IPDI contents of 11% (a) and 13% (b).
